# The involvement of rare disease patient organisations in therapeutic innovation across rare paediatric neurological conditions: a narrative review

**DOI:** 10.1186/s13023-022-02317-6

**Published:** 2022-04-18

**Authors:** Christina Q. Nguyen, Kristine Alba-Concepcion, Elizabeth E. Palmer, Jackie L. Scully, Nicole Millis, Michelle A. Farrar

**Affiliations:** 1 Discipline of Paediatrics and Child Health, School of Clinical Medicine, UNSW Medicine and Health, UNSW Sydney, Sydney, NSW Australia; 2 Department of Neurology, Sydney Children’s Hospital Network, Sydney, NSW Australia; 3 Centre for Clinical Genetics, Sydney Children’s Hospital Network, Sydney, NSW Australia; 4 Disability Innovation Institute, UNSW Sydney, Sydney, NSW Australia; 5 Rare Voices Australia, Melbourne, VIC Australia

**Keywords:** Advanced therapeutics, Paediatric neurology, Patient advocacy, Precision medicine

## Abstract

**Background:**

The patient voice is becoming increasingly prominent across all stages of therapeutic innovation. It pervades research domains from funding and recruitment, to translation, care, and support. Advances in genomic technologies have facilitated novel breakthrough therapies, whose global developments, regulatory approvals, and confined governmental subsidisations have stimulated renewed hope amongst rare disease patient organisations (RDPOs). With intensifying optimism characterising the therapeutic landscape, researcher-advocate partnerships have reached an inflexion point, at which stakeholders may evaluate their achievements and formulate frameworks for future refinement.

**Main text:**

Through this narrative review, we surveyed relevant literature around the roles of RDPOs catering to the rare paediatric neurological disease community. Via available literature, we considered RDPO interactions within seven domains of therapeutic development: research grant funding, industry sponsorship, study recruitment, clinical care and support, patient-reported outcome measures, and research prioritisation. In doing so, we explored practical and ethical challenges, gaps in understanding, and future directions of inquiry. Current literature highlights the increasing significance of ethical and financial challenges to patient advocacy. Biomedical venture philanthropy is gaining momentum amongst RDPOs, whose small grants can incrementally assist laboratories in research, training, and pursuits of more substantial grants. However, RDPO seed funding may encounter long-term sustainability issues and difficulties in selecting appropriate research investments. Further challenges include advocate-industry collaborations, commercial biases, and unresolved controversies regarding orphan drug subsidisation. Beyond their financial interactions, RDPOs serve instrumental roles in project promotion, participant recruitment, biobank creation, and patient registry establishment. They are communication conduits between carers, patients, and other stakeholders, but their contributions may be susceptible to bias and unrealistic expectations.

**Conclusion:**

Further insights into how RDPOs navigate practical and ethical challenges in therapeutic development may enhance cooperative efforts. They may also inform resources, whose distribution among advocates, parents, and clinicians, may assist decision-making processes around rare disease clinical trials and treatments.

## Background

According to its widely accepted definition, a rare disease (RD) is a condition affecting no more than 5 in 10,000 people [1]. Uncommon as they are individually, RDs have a global point prevalence of 262.9–446.2 million [2]. Of 7000–8000 distinct RDs, approximately 72–80% have genetic aetiologies, 75% demonstrate neurological symptoms, and 70% have exclusively paediatric onset [2, 3]. Though the health needs of RD patients are often unmet, their potential for refinement offer an impetus for academic research [4, 5].

**Fig. 1:**
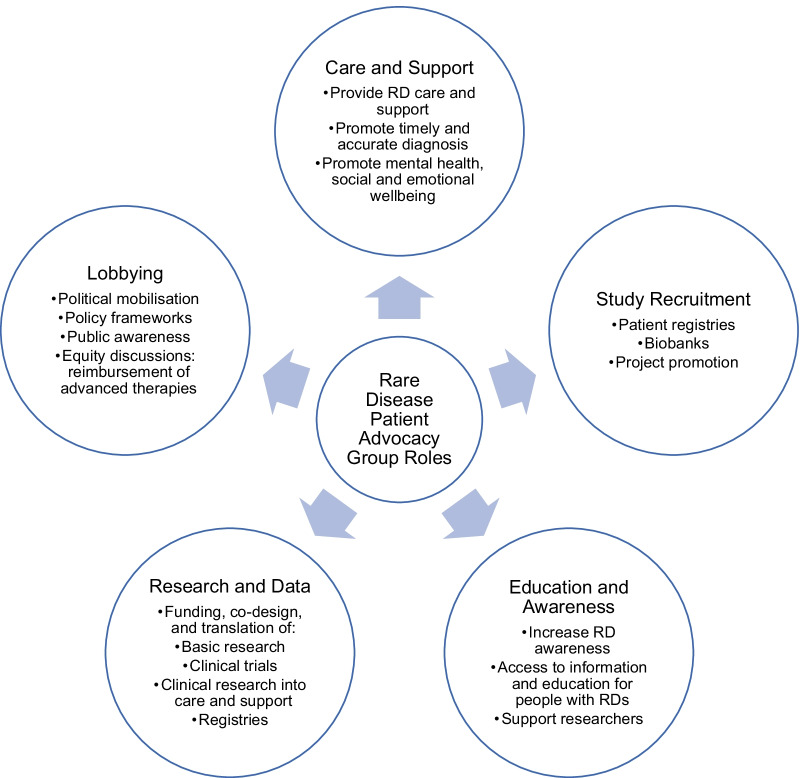
Rare disease patient organisation roles and responsibilities in therapeutic innovation (information from [14, 15, 21, 23])

RD research has historically been neglected due to its limited target markets, patient renumeration capacities, and treatment profitability [6]. Across East and South–East Asia, insubstantial governmental and financial supports hamper patients’ transitions from research participants to equal collaborators, especially in the field of drug research and development [7, 8]. Nonetheless, some countries have observed significant progress in RD consumer engagement. By offering pharmaceutical manufacturers 7 years of marketing exclusivity, the United States of America Orphan Drug Act 1983 incentivised RD-directed clinical research and inspired similar laws internationally [6, 9]. With this alleviation of economic pressure, RD research has undergone a paradigm shift from academic and commercial interest, to scientific ‘democratisation,’ and community engagement [10, 11]. Australia’s National Health and Medical Research Council (NHMRC) Act 1992 recognises public consultation as an integral aspect of research, such that its guidelines for grant review value outcome significance over disease prevalence [12]. The Act mandates meaningful consumer engagement throughout all research stages; plans for consumer consultation evidenced in grant applications; and routine appointment of patient representatives to principal, advisory, and peer review committees [10, 13]. RD patient organisations (RDPOs) are active in biomedical discourse, at least in North America, Europe, and Australia [14]. While waging *bella contra morbum*, war against disease, they increasingly engage with researchers through sponsorship, recruitment, and logistical support [11]. They respond to extensive patient and clinician unmet needs—including awareness, care, support, and timely, accurate diagnoses—all of which are as essential as therapeutic innovation to achieving wellbeing for families living with RDs (Fig. 1) [4, 15].

Advances in genomic diagnostic and therapeutic technologies facilitate disease identification and novel treatment development. They potentially offer breakthrough therapies for many RDs, including neuromuscular and neurometabolic conditions like spinal muscular atrophy (SMA) and Batten disease [16, 17]. Evidently, the development, regulatory approval, and restricted government subsidisation of SMA drug, nusinersen (Spinraza), and late-infantile Batten disease therapy, cerliponase alfa (Brineura), in Australia and other countries has stimulated hope amongst RDPOs [18, 19]. With optimism and investment intensifying across the therapeutic landscape since the turn of the last decade, researcher–advocate partnerships are becoming increasingly prominent at all stages of therapeutic innovation [20, 21]. While RDPOs may serve as subjective vessels for the patient experience, especially around therapeutic impact, they may be under-informed regarding complex drug development [11, 21]. Through a narrative review of the literature, we considered RDPOs and their interactions within several domains of therapeutic development. In doing so, we explored the following critical areas of engagement: grant funding; financial support from pharmaceutical companies; study recruitment; patient care and support; patient reported outcomes and experience measures; and research priorities. We present a review on patient advocate contributions to paediatric advanced neurotherapeutics, exploring ethical challenges, practical difficulties, and future directions of inquiry regarding RDPOs and their ‘public shaping’ of biomedical research [22]. This narrative review’s methodology is detailed in the ``Appendix’’.

## Main text

### Grant funding

Many advocacy foundations are adopting biomedical venture philanthropy models [24]. In the absence of industry funding, venture philanthropy secures small funds for under-financed stages of therapeutic development, incentivises research, and reduces the risk inherent in novel therapy commercialisation. In their mixed methodology study, Pinto et al. [21] identified research as a priority for almost three quarters of surveyed Australian RDPOs, with 59% having funded research within the preceding 5 years. Most RDPOs make financial contributions in the five to mid-six figure range (USD) per fiscal year [21, 25]. While relatively small, such funds—as Panofsky [26] notes—assist laboratories incrementally in their research, training, and pursuit of more substantial grants. Indeed, the Tourette Syndrome Association of America awarded $21 million to over 450 projects from 1984 to 2016, with every $1 of funding since 2003 corresponding to $10 of government sponsorship in the next phase of work [27]. Seed funding may encounter long-term sustainability and efficiency difficulties [28]. Even so, private funding may promote siloed research and limit research capacity and collaboration [29, 30].

Several studies describe potential difficulties associated with RDPO research fund allocation. Specifically, they identify poorly informed grant assignment, use of ‘lay expertise’ for decision-making, and insufficient communication between scientists and patient representatives regarding expectations as potential issues [11, 21, 26]. Taken together, these factors may compound difficulties assessing research investments for clinical impacts beneficial to people with RDs. In some RDPOs, they may also highlight the insufficiency of governance mechanisms, which would otherwise guarantee accountability from beneficiaries of RDPO grants and ad hoc funding [26].

By allocating funding specifically to RD research, public initiatives may secure financial backing without forfeiting the rigour of grant submission peer review processes [31]. For instance, over £3 million of research and development funding is designated to a centralised biobank of tissue and biofluid resources in the United Kingdom [32]. The NHMRC also extends targeted and urgent calls for research, serving to stimulate research or research capacity in focused health and medical science fields and guarantee funded research into RD objectives [33].

According to Dear et al. [31], dedicated funding for ultra-orphan drug development raises concerns around the cost-effectiveness and commercialisation of approved therapies, with pharmaceutical companies earning large profits, consumers accruing high costs, and patients encountering treatment access barriers. However, Chapman et al. [34] argue that applying traditional cost-effectiveness analyses to funding assessments of RD treatments may artificially inflate opportunity costs and underestimate therapeutic impacts on healthcare, society, and people with RDs. They propose two models to effectively lower market prices: (1) distributing the economic benefits between the innovator/industry and healthcare sector, and (2) capping the number of years during which the innovator is assigned 100% profits. Incorporating lower market prices into cost calculations enables a more realistic comparison with current treatments [35]. Further research into methods of assesing RD treatment affordability and sustainability is needed to inform future public funding decisions, especially around patient-centred outcomes.

### Financial support from pharmaceutical industries

Australia’s National Strategic Action Plan for Rare Diseases (henceforth, Australia’s Action Plan) highlights the importance of commercial research grants independent of governmental and volunteer-out-of-pocket funding in providing financial continuity, a sustainable workforce, and effective operation [36]. This is significant since many RDPOs are run by volunteers, parents, or specific RD communities [21]. Between RDPOs and biopharmaceutical companies, Stein et al. [37] recognise a consistent need to establish metrics for evaluating collaborations. While acknowledging that guidelines can help ensure appropriate fund use and inter-stakeholder communication, they extend on previous studies by recommending transparency and guidance vis-à-vis RDPOs’ receipt of pharmaceutical industry funds. A systematic review on patient advocacy groups non-specific to RD by Fabbri et al. [38] identifies the commonality of industry funding, citing an estimated prevalence of up to 83% and possible issues around disclosure of potential conflicts of interest. Limited studies suggest a probable increased likelihood among pharmaceutical companies to collaborate with consumer groups of aligned interests [38]. However, instances of specific RDPO conflicts of interest are not well-documented in the literature. Considering that industry-funded RDPOs may be better resourced and positioned to exert influence over health policy, such commercial sponsorships may skew representations of patient interests [38].

Industry intervention may introduce commercial biases into the representation of patient interests, misaligning public health priorities and advocacy-driven policies [39]. This is important considering the educational and recruitment roles that advocacy groups adopt amongst their communities [39–41]. Koay and Sharp [42] suggest that certain collaborations with for-profit companies can damage reputations, limit community support, and restrict future partnerships [43]. Recognising these concerns, Stein et al. [37] recommend that RDPOs exclude representatives of biopharmaceutical companies actively developing or marketing treatments for their RD from directorial boards.

### Study recruitment

The sparse numbers and geographical distributions of people with RD can represent considerable research barriers. Studies highlight the unique ability of RDPOs to accumulate resources for researchers, commending RDPO support in project promotion, active participant recruitment, biobank creation, and patient registry establishment [26, 42, 44]. When patient registries are advocate-led, they typically gather larger cohorts, and greater genetic and clinical diversity [44]. However, consumer-led patient registries may overlook internal validity issues and not comply with national or international standards. An global survey of myotonic dystrophy registries revealed that 41% failed to collect all mandatory items cited by the “Naarden” dataset criteria—an international minimal core dataset for myotonic dystrophy registries established in 2009 by key opinion leaders [45–47]. Contributing impediments potentially included miscoding, ambiguous inclusion criteria, and difficulties with quantifying external completeness—that is, the degree to which all cases were captured [48]. Panofsky [26] identifies overly restrictive consent requirements as potential barriers to utility and researcher appeal. Likewise, Denton et al. [49] associates most proprietary databases with redundant data, restricted researcher access, and inadequate meaningful outcome and natural history information. Nonetheless, logistical challenges with accessing phenotypic and genotypic data from affected individuals create opportunities for RDPO involvement [50]. Among academics, there exists a consensus that clinical registries, when dually researcher- and RDPO-informed, can accrue edifying natural history data and advise study design [51, 52].

Recent studies support the catalytic effect of researcher-RDPO partnerships on trial recruitment and retention. Peay et al. [53] surveyed 203 carers of children with SMA, Duchenne muscular dystrophy (DMD), or Becker muscular dystrophy, and found that parental concerns regarding children’s potential randomisation to placebo arms tempered interests in paediatric neuromuscular trials. To navigate these challenges, Bartlett et al. [54] designed multimedia tools in consultation with RDPOs: indeed, they consulted Cure SMA and incorporated its feedback into an SMA recruitment/retainment study plan. Their RDPO-informed tool development and promotion achieved a 40% recruitment rate among eligible Cure SMA registrants [54]. Large-scale RDPOs have become increasingly proactive in trial recruitment, mobilising specialists, patient groups, and industry representatives towards a common cause. For instance, TREAT-NMD has a designated advisory committee for therapeutics [55]. Their review board engages with pharmaceutical companies and regulatory affairs, ensuring the realisation of translational research. TREAT-NMD also maintains patient registries aimed at enabling clinical trial feasibility [55]. Moreover, the European Organisation for Rare Diseases (EURORDIS), a non-profit alliance of 984 RDPOs from 74 countries, plays critical roles in consumer engagement, particularly within cross-national clinical research structures like the European Reference Networks [56]. Together, their platforms establish a sustainable ecosystem, wherein research, care, and medical innovation are in communication and collaboration, thus benefitting RD patients and communities [55, 56]. Such large-scale RDPOs are examples of how internal validity and governance protocols may support collaborative study recruitment.

### Care, support, and therapeutic access

According to a qualitative study in Australian mothers of children with SMA, financial, opportunity, and psychosocial caregiving costs affect all elements of family wellbeing, from career changes to biopsychosocial distress [57]. Some carers attribute their timely access to funding, equipment, and community assistance, to RDPO intervention [57]. Studies in other rare neurological disorders, such as childhood-onset developmental and epileptic encephalopathies, have reported similar findings [58]. By offering financial assistance, educational tool development, service coordination, fostered community solidarity, and professional healthcare training, RDPOs may facilitate the implementation of new therapies [15, 59]. In fact, their assistance with patient care, support, and therapeutic access may help optimise outcomes [15, 59]. Even in overstretched RDPOs, patient education, family consultation, and community gatherings remain achievable priorities when addressing unmet patient needs [7].

While constructing a 2015 policy framework for RD therapeutic development, Menon et al. [60] reported findings from interviews of patient communities across Canada and identified prescription drug coverage as a leading concern. They reported themes of low patient engagement in reimbursement reviews, non-transparent decision parameters, and seemingly biased appeal processes. A systematic review by Short et al. [23] supports these concerns, highlighting how RDPOs may help prioritise clinical (rather than financial) considerations at reimbursement panels. Notably, consumer and RDPO participation throughout formal regulatory and reimbursement processes is increasing. As patient-perceived therapeutic benefits may differ from measured clinical trial endpoints, they may inform pertinent discussions around cost–benefit analyses, subsidisation, and reimbursement [61]. Nonetheless, they have yet to be recognised in conventional value assessments.

Regarding the roles that RDPOs may assume in facilitating therapeutic access, there remains active academic discourse. Alongside their meaningful contributions to approval and reimbursement processes, RDPOs may advise pharmaceutical companies on managed access programs, enabling treatment prior to local approval, treatment subsidisation, and advocacy [62]. Future studies should seek clarification and explore how RDPOs perceive their lobbyist roles in drug marketing and equitable patient access.

### Patient reported outcome and experience measures (PROMs, PREMs)

Interventional studies increasingly focus on PROMs and PREMs, which may help align patient and researcher perceptions of clinically meaningful improvements [63]. Consumer engagement helps researchers appreciate patient and family experiences in healthcare and research, promoting outcomes of the highest consumer-held priorities [51]. As documented in various studies, patients and advocates commonly value research relevance enabled by patient input, with the adoption of new therapies dependent on perceived acceptability (Table 1) [26, 60, 64, 65]. Indeed, Australia’s Action Plan encourages researcher–consumer collaborations and establishes lived patient experiences as the basis for all research [36].

**Table 1:** Quotes from RD patient representatives justifying their consultation throughout therapeutic development (information from [60, 64, 65])

Author(s)	Study type	Cohort	Speaker	Quote
Menon et al. [60]	Mixed methods	Patient communities	Unnamed	“I think the patient is critical, and the caregiver, to put a framing around what that means to them versus just the hardcore data”
Gaasterland et al. [64]	Qualitative	Patient Think Tank (PTT) members of ASTERIX project	PTT member	“Yeah, I suppose that’s the key thing really, is making sure that patients have the chance to give their views, and then that those views are listened to. And…kind of more practical things. They…the patients wanted to make sure that…the kind of outcomes were sort of relevant in their life, so, you know, the idea of looking beyond just the clinical outcomes”
Morel et al. [65]	Mixed methods	Patients	Patients	(1) “Anything that engages the sufferer in discussions determining how to handle treatment/medication can only be beneficial to the patient”
				(2) “But ‘minor’ side effects can be extremely wearing and challenging when they occur every day. Mental side effects are very difficult to manage”

In translating clinical trial data to meaningful everyday outcomes, RDPOs serve a bidirectional role. Firstly, they may offer a comprehensive understanding of outcomes beyond clinical outcome measures and inform therapeutic benefits, acceptability, and expectations [66]. In partnership with other stakeholders, RDPOs can also inform clinical protocols defining ‘contexts of use’ (e.g. eligibility) and ‘concepts of interest’ (e.g. function) [67, 68]. Klingels et al. [69] consulted data collected by the Duchenne Parent Project, a Netherlands-based RDPO, to develop a DMD PROM for upper limb function. This not only reflects a shift from the hitherto commonly employed 6-minute walk test to upper limb motor assessments, but also, captures the potential for systemic remodelling based on advocate feedback [68].

Secondly, RDPOs can advise patients on the relevance of risk, benefit and outcome data to their lives [70]. Optimising current technologies, RDPOs have adopted online platforms to present research in accessible forms with broad potential reach [20, 71]. In their survey of 124 genetic organisations, Landy et al. [25] reported that 110 groups disseminated research findings via their websites. The International Dravet Epilepsy Action League (IDEAL) remains a guiding exemplar, using websites, chatrooms, and linguistically diverse subforums to support informed decision-making among parents of affected children [59]. Such studies suggest that internet-based social networks help RDPOs establish community understanding around how advanced therapies can modify quality of care [25, 59, 70].

### Research priorities

Historically, relationships between scientists and advocacy leaders encounter varying degrees of tension, with conflicting research priorities serving a common source [42]. Previously documented sources of friction include researcher concerns surrounding the devaluation of basic research, and fixation on cure observed among some advocacy group leaders [72]. Provided they work closely with researchers, RDPOs may wield the authority to intervene and commandeer the course of innovation [26]. Some studies praise this disruption of scientific autonomy as a means by which RDPOs overcome their marginalisation [26]. By aligning research and patient agendas, RD advocacy groups can facilitate protocol feasibility and success [20].

Nonetheless, these studies overlook the feasibility of demands for immediately applicable clinical breakthroughs and sometimes limited support ascribed to basic research. Pinto et al. [11] categorised 15% of interviewed RDPO leaders as “passionate cure-seekers” and detailed a shared concern amongst other participants that those over-invested in cures could overlook quality-of-life, patient dignity, and interpersonal relationships. Another paper reported that small RDPOs devoted to conditions with little prospect of cure in the foreseeable future were least likely to prioritise research [21]. Realising these potential complications, some RDPOs have broadened their ambit to include symptomatic and psychological relief [11, 26]. This area is pertinent considering the oftentimes debilitating nature of RDs [73].

Among advocacy groups, there exists scope for improvement in efficiency and standardisation in order to meaningfully set and address research priorities. Interview-based studies demonstrate a positive correlation between organisational size and research contributions, noting that small, under-resourced groups often struggle to achieve research goals, due to high workloads, administrative costs, and replicative efforts [7, 21]. In a rare epilepsy landscape analysis, seven organisations deemed their patient registries unsustainable, raising possible issues around lack of expertise in database maintenance and utilisation [74, 75]. Financial unsustainability may also restrict the breadth of support and research services that individual RDPOs can offer. According to Li et al. [7], none of 28 interviewed Chinese RDPOs had reliable funding sources or sponsored academic research. In fact, only five (17.9%) and 13 (46.4%) had assisted with clinical trial recruitment and registry establishment, respectively [7]. Conceivably, cross-collaboration, with appropriate government endorsement, can strengthen patient empowerment and sustainable long-term goals [7, 75].

In the context of ‘silo mentalities’, data sharing is complicated by commercial conflicts of interest and concerns around forfeiting professional advantages secured through data ownership. However, limitations in data sharing hinder reciprocal operations between institutions [76]. Insular data management and inter-organisational competition may force the ‘reinvention of the wheel’ [29, 30]. While multiple organisations may support a single RD, collaborative efforts are critical to keeping RD communities at the centre of research progress [75].

To many RDPOs, RD registries remain a notable research prioritiy. However, siloed efforts by small RDPOs may contribute to pitfalls of external completeness. An interview-based study reported that, of ten RDPO-run registries, the largest two still represented fractions of patients nationally, despite high absolute numbers [9]. Umbrella organisations, such as TREAT-NMD, EURORDIS, and RVA, can unify individual foundations and mobilise advocacy around establishing national RD registries and alliances [9, 77]. As Lacaze et al. [77] propose, a national RD registry would address consistency and efficiency concerns across registries, information platforms, and governance and consent protocols. It would apply the F.A.I.R. (Findability, Accessibility, Interoperability, Reusability) Guiding Principles for Scientific Data Management and Stewardship, standardising language, facilitating meta-analyses, and enhancing research interoperability [78]. Previous qualitative research with patients, family members, and carers supports this recommendation, suggesting that data harmonisation from established parameters improves sustainability [60]. Likewise, Australia’s Action Plan supports a national registry [36].

Recent studies recommend further investment in international collaborative infrastructure to support therapeutic innovation [77, 79, 80]. Arguably, multinational, multidisciplinary integration enables multicentre clinical trial readiness; this, when combined with transnationally curated registry data, may maximise the collective impact of global research expenditure [77, 79, 80]. Umbrella organisations can assist by pooling resources and training patient advocates at the national/transnational level [9, 70, 77]. Indeed, RVA and EURORDIS represent 90 and 984 RDPOs, respectively, and assist their partners in patient advocacy through educational resources [81–83]. Further study of how RDPOs interact and navigate disease-specific challenges may enhance cooperative efforts at the individual, umbrella, and consortium levels.

The past decade has observed a steady increase in therapeutic development, especially pertaining to rare paediatric neurological diseases [84]. Alongside this proliferation of biomedical technologicals, the focus of academia has shifted towards patient-centred practices, with RDPOs increasingly recognised as active stakeholders [85]. Given this rapidly evolving landscape, we deemed a narrative review methodology best-suited for broadly understanding progress and future directions of inquiry. Our search strategy suggested that there were limited peer-reviewed articles examining the dynamic roles of RDPOs in therapeutic innovation for rare paediatric neurological diseases. However, as a narrative review, this article does not include all relevant studies, potentially leading to selection bias. As we included studies published in English, we may have overlooked informative studies published in other languages. Most included articles were from high-income countries, possibly limiting generalisability. A future systematic review to identify, select, and critically appraise relevant primary research, and extract and analyse data, may provide further insights into this rapidly changing field.

## Conclusions

Bidirectional communication between patients and other stakeholders is becoming increasingly critical to the success of therapeutic innovation, with various umbrella organisations publishing guidelines and policy frameworks around RD research [36, 86]. Paediatric neurology RDPOs serve as communication conduits between parents, patients, and other stakeholders, shaping research design and recruitment based on parental concerns and priorities [69, 87–89]. Nonetheless, there remains controversy around these RDPOs’ use of industry funding. Current literature highlights the growing significance of ethical and economic concerns to patient advocacy, supporting the argument that ethicists and economists should be consulted alongside advocates and implementation scientists from project onset. However, to our knowledge, original research into advocate experiences of paediatric neurological RDs (and RDs generally) remains limited, with most literature comprising position statements, protocols, and review articles [14]. RDs of paediatric neurological subtypes face their own unique challenges in patient support and therapeutic development [51]. There is an urgent need for further original research into the experiences and expectations of patient advocates regarding therapeutic development. Such insights may later inform a decision-making framework to guide interactions between researchers and RDPOs [90].

## Data Availability

Not Applicable.

## Appendix: Narrative review methodology

This narrative review was conducted using various search terms, such as “rare disease,” “neurological disease,” “patient advocacy,” and “consumer organisation” within several databases—namely, MEDLINE, PubMed, and Embase. This enabled the identification of many relevant, pivotal studies without a specified systematic protocol or pre-defined analysis approach. RDPO websites were also reviewed and incorporated into the reference list. Peer-reviewed books, commentaries, editorials, and reports were included when they related to RDPOs broadly or RDPOs with a neurological focus. Published articles focused on English language articles and seminal or influential papers. The findings from these sources were integrated into our review as appropriate. As a narrative review, authors’ assumptions and biases may be a limitation later addressed by a systematic review in this rapidly changing field.

## Abbreviations

DMD: Duchenne muscular dystrophy
EURORDIS: European Organisation for Rare Diseases
IDEAL: International Dravet Epilepsy Action League
NHMRC: National Health and Medical Research Council
PREM: Patient reported experience measure
PROM: Patient reported outcome measure
RD: Rare disease
RDPO: Rare disease patient organisation
RVA: Rare Voices Australia
SMA: Spinal muscular atrophy
